# Patent foramen ovale closure in non-elderly and elderly patients with cryptogenic stroke: a hospital-based cohort study

**DOI:** 10.3389/fneur.2023.1190011

**Published:** 2023-05-16

**Authors:** Po-Lin Chen, Chi-Sheng Wang, Jin-An Huang, Yun-Ching Fu, Nien-Chen Liao, Chiann-Yi Hsu, Yu-Hsuan Wu

**Affiliations:** ^1^Division of Neurology, Taichung Veterans General Hospital, Neurological Institute, Taichung, Taiwan; ^2^School of Medicine, Institute of Brain Science, National Yang Ming Chiao Tung University, Taipei, Taiwan; ^3^Department of Post-Baccalaureate Medicine, College of Medicine, National Chung Hsing University, Taichung, Taiwan; ^4^Department of Health Business Administration, Hungkuang University, Taichung, Taiwan; ^5^Department of Pediatrics, Children's Medical Center, Taichung Veterans General Hospital, Taichung, Taiwan; ^6^Department of Pediatrics, School of Medicine, National Chung Hsing University, Taichung, Taiwan; ^7^Department of Pediatrics and Institute of Clinical Medicine, National Yang Ming Chiao Tung University, Taipei, Taiwan; ^8^Department of Critical Care Medicine, Taichung Veterans General Hospital, Taichung, Taiwan; ^9^Biostatistics Task Force, Taichung Veterans General Hospital, Taichung, Taiwan

**Keywords:** cryptogenic stroke, ischemic stroke, outcome, patent foramen ovale, patent foramen ovale closure

## Abstract

**Introduction:**

The efficacy of patent foramen ovale (PFO) closure in the elderly population is unclear. We aimed to investigate the efficacy and safety of PFO closure in non-elderly and elderly patients.

**Methods:**

Patients over 18 years of age with cryptogenic stroke (CS) or transient ischemic attack and PFO were prospectively enrolled and classified into two groups according to treatment: (1) closure of PFO (the PFOC group) and (2) medical treatment alone (the non-PFOC group). The primary outcome was a composite of recurrent cerebral ischemic events and all-cause mortality during the follow-up period. A modified Ranking Scale [mRS] at 180 days was recorded. The safety outcomes were procedure-related adverse events and periprocedural atrial fibrillation. The results between the PFOC and non-PFOC groups in non-elderly (<60 years) and elderly (≥60 years) patients were compared.

**Results:**

We enrolled 173 patients, 78 (45%) of whom were elderly. During a mean follow-up of 2.5 years, the incidence of primary outcome was significantly lower in the PFOC group (6.2% vs. 17.1%, hazard ratio[HR] = 0.35, 95% CI 0.13–0.97, *p* = 0.043) in adjusted Cox regression analysis. Compared with the non-PFOC group, the PFOC group had a numerically lower risk of the primary outcome in both the elderly (HR 0.26, 95% CI 0.07–1.01, *p* = 0.051) and the non-elderly (HR 0.61, 95% CI 0.11–3.27, *p* = 0.574) groups. In addition, patients with PFO closure in the elderly group had a lower median mRS at 180 days (*p* = 0.002). The rate of safety outcome was similar between the non-elderly and elderly groups.

**Discussion:**

PFO closure was associated with a reduced risk of the primary outcome in patients with PFO and CS in our total cohort, which included non-elderly and elderly patients. Compared to those without PFO closure, elderly patients with PFO closure had a better functional outcome at 180 days. PFO closure might be considered in selected elderly patients with PFO.

## 1. Introduction

Patent foramen ovale (PFO), which may cause paradoxical embolism, has been proposed to have a possible causal association with cryptogenic stroke (CS) (1–3). Transcatheter closure for PFO may prevent recurrent stroke in selected patients with CS (4).

Recent randomized trials and global guidelines for PFO closure focused on patients younger than 60 years with high-risk anatomical features, including large shunt size and atrial septal aneurysm (ASA) (3–5). Although the prevalence of PFO is 34–66% in elderly patients with CS, (1) the causal role of PFO is often overlooked due to multiple competing low-medium risk potential causes of index stroke. However, PFO is not only associated with CS in elderly patients, (6) but increasing age is also associated with a higher risk of recurrent ischemic stroke in patients with PFO and CS or cryptogenic transient ischemic attack (TIA) (7, 8). Therefore, elderly patients with PFO could be more susceptible to paradoxical embolism. PFO closure might reduce recurrent strokes and improve long-term outcomes in carefully selected elderly patients with PFO and CS. However, there is still concern about the efficacy and higher risk of periprocedural complications in elderly patients (7–11).

Previous large-scale studies tend to postpone the procedure to 80–100 days after the onset of acute stroke (3, 5) possibly due to a required monitoring period of potential atrial fibrillation. However, global guidelines for PFO closure have not provided a precise time point to perform PFO closure (4). Therefore, the adequacy of performing PFO closure in the acute stage is unclear.

We hypothesized that the elderly patients (≥60 years) with CS and PFO might also benefit from PFO closure as the non-elderly group (<60 years). In addition, the efficacy and safety of PFO closure in the acute stage were assessed.

## 2. Materials and methods

### 2.1. Study design and participants

The study is a hospital-based cohort study from a prospective stroke registry at a comprehensive stroke center in central Taiwan. The study was approved by the Institutional Review Board (IRB) of Taichung Veterans General Hospital (CG19339B). Between January 2013 and October 2021, patients with acute ischemic stroke (AIS) or TIA were screened. Inclusion criteria were patients with (1) CS or cryptogenic TIA, (2) PFO, and (3) aged over 18 years. Exclusion criteria were (1) patients who were diagnosed with pulmonary arteriovenous malformation (AVM) according to the transcatheter procedure and (2) a follow-up period of <6 months. The IRB of Taichung Veterans General Hospital granted a waiver for consent.

Patients were classified into two groups: (1) the PFO closure group (PFOC) included patients who underwent transcatheter PFO closure in addition to medical therapy and (2) the non-PFO closure group (non-PFOC) included patients who received medical therapy alone. Demographic characteristics and ancillary tests at admission were recorded, and telephone questionnaires were performed during the follow-up period. The primary outcome was a composite of recurrent cerebral ischemic events and all-cause mortality during the follow-up period. The secondary outcome was the modified Rankin Scale (mRS) at 180 days and a favorable outcome at 180 days, defined as an mRS score of 0–2. The follow-up period of each patient ended when (1) there was a recurrent cerebral ischemic event, (2) mortality of any cause, or (3) loss of follow-up. Procedure-related adverse events and new-onset periprocedural atrial fibrillation (AF) that occurred within 7 days after the procedure were recorded. The results between the treatment groups were compared in the non-elderly (<60 years) and elderly (≥60 years) groups, respectively.

### 2.2. Assessment of cryptogenic stroke or TIA

All ischemic strokes were classified according to the criteria from the Trial of ORG 10172 in the acute stroke treatment study (12). Only patients with unilateral weakness for more than 10 min were diagnosed with TIA. In our hospital, a routine survey of stroke etiologies includes brain computed tomographic angiography or magnetic resonance angiography, ECG, transcranial color-coded duplex sonography, and duplex sonography of cervical arteries. We performed further studies, including autoimmune disease, coagulopathy, hereditary diseases, malignancy, 24-h Holter ECG, and transthoracic echocardiography when a routine survey did not reveal an attributable stroke etiology. When an etiology or mechanism for the index ischemic event could not be identified, a microbubble test was performed to detect the presence of PFO. Patients with an abnormal microbubble test were assumed to have PFO. CS or cryptogenic TIA was diagnosed after excluding identifiable etiologies or mechanisms other than PFO.

### 2.3. Assessment of PFO and risk classification

Patent foramen ovale was evaluated using a microbubble test based on gaseous contrast transcranial Doppler (TCD) ultrasonography focusing on the unilateral middle cerebral artery. In brief, agitated saline with an additional 1 ml of the patient's blood and 1 ml of air was injected intravenously as a contrast agent while the Valsalva maneuver was being performed (13). The grading of shunt size was based on the maximum number of microbubble signals on the TCD spectrum within 30 s from contrast injection. Microbubble tests were graded as normal for 0 microbubble signal, small for 1–5, moderate for 6–25, and large for >25 according to a previously validated method (3). The Risk of Paradoxical Embolism (RoPE) score was used to assess the potential risk of stroke from PFO. A higher RoPE score indicates a higher probability that an observed PFO is pathogenically related to CS (14).

### 2.4. Transcatheter PFO closure

All patients diagnosed with CS and PFO were eligible for PFO closure unless there was active bleeding, allergy to radiographic contrast, acute pulmonary edema, or active systemic infection. The multidisciplinary stroke team would discuss the potential benefits and risks of PFO closure with the family or patient in a shared decision-making conference. Cardiac catheterization for diagnosis and closure could proceed with the consent of the patients and their families.

The procedure of the implantation of the PFO occluder was the same as in previous studies (15). All patients, whether they had closed PFO or not, received antithrombotic therapy according to the clinical guidelines (4, 16). Antiplatelet regimens included aspirin (100 mg once daily) or clopidogrel (75 mg once daily) alone and dual antiplatelet therapy. Oral anticoagulant (warfarin or non-vitamin K antagonist oral anticoagulant) would be administered to patients diagnosed with periprocedural AF.

### 2.5. Statistical analyses

Continuous data were presented as mean ± standard deviation (SD) and categorical data as numbers with percentages, while discrete variables were expressed as the median and interquartile range (IQR). We used Fisher's exact test or the X^2^ test to analyze categorical variables, while continuous variable analyses were performed using the Mann–Whitney U-test. A *p*-value <0.05 was considered to be statistically significant. Cox regression analyses adjusted by the RoPE score, and NIH Stroke Scale (NIHSS) at admission were performed to investigate the effect of PFO closure on the incidence of primary outcomes compared to non-PFOC. Adjusted hazard ratio (HR) with a 95% confidence interval (CI) was calculated accordingly between the PFOC and non-PFOC groups. The occurrence rate of primary outcomes was compared using the log-rank test and plotted with the Kaplan–Meier method to highlight the time-to-event analyses. We also performed subgroup analyses using adjusted Cox regressions and then plotted using the forest plot according to HR. Binary logistic regression was performed for analyzing the effect of PFO closure on the favorable outcome.

## 3. Results

### 3.1. Baseline characteristics between the PFOC and non-PFOC groups

**Figure 1:**
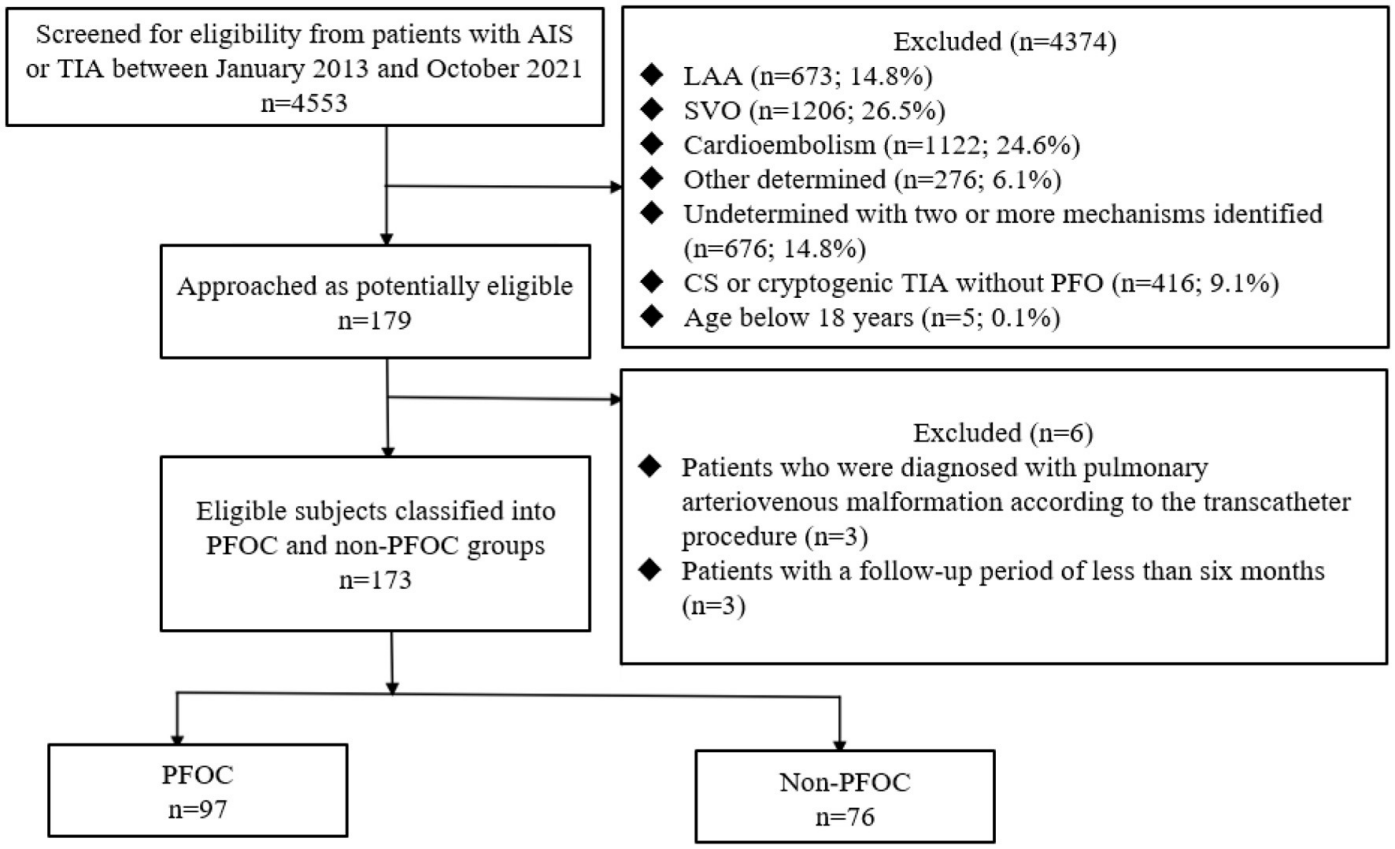
Flow diagram. AIS, acute ischemic stroke; TIA, transient ischemic attack; LAA, large artery atherosclerosis; SVO, small vessel occlusion; CS, cryptogenic stroke; PEOC, patent foramen ovale closure.

A total of 179 patients with CS and cryptogenic TIA were diagnosed with PFO (Table 1). Three patients diagnosed with pulmonary AVM on transcatheter examination and three with a follow-up period of <6 months were excluded (Figure 1). Therefore, 173 patients were eligible for this study. In the total cohort, the mean age (SD) was 56.5 (14.9) years, and 78 patients (45.1%) were 60 years or older (Table 1). The mean follow-up period (SD) was 2.5 (1.7) years. Most patients had AIS (91.9%) with a median NIHSS score (IQR) of 2 (1–3). The size of the PFO shunt was moderate to large in 100 patients (57.8%). The mean RoPE score (SD) was 5.5 (1.9), and 84 patients (48.6%) had a RoPE score ≥6. A total of 97 (56.1%) and 76 (43.9%) patients received PFO closure and medical therapy, respectively. Compared to the non-PFOC group, the PFOC group was younger (mean age [SD] 53.6 [14.4] vs. 60.2 [14.9] years, *p* = 0.004), had fewer elderly with ≥60 years (36.1% vs. 56.6%, *p* = 0.011), less diabetes (15.5% vs. 35.5%, *p* = 0.004), and a longer mean follow-up period (2.9 [1.7] vs. 2.1 [1.5] years, *p* = 0.001). Furthermore, the PFOC group had a higher frequency of moderate to large PFO shunt (80.4% vs. 29.0%, *p* < 0.001) but a similar frequency of ASA (1.0% vs. 1.3%, *p* = 1.000). The PFOC group had a higher mean RoPE score (5.9 [1.8] vs. 5.0 [2.0], *p* = 0.001), and more patients had a RoPE score of ≥6 (55.7% vs. 39.5%, *p* = 0.050). The median NIHSS score and antithrombotic strategies were comparable between the PFOC and non-PFOC groups. The median time from the index event to the microbubble test was 9 days (IQR 7.3–21.9) and was similar between the groups. The median time from the microbubble test to PFO closure in the PFOC group was 4 days (IQR 3.65–21.9). Thus, the median days from the index stroke to PFO closure were 13 days (IQR 10.5–35.5).

**Table 1:** Baseline characteristics of patients with PFO closure and non-PFO closure.

**Characteristics**	**Total (*n* = 173)**	**PFOC (*n* = 97)**	**Non-PFOC (*n* = 76)**	***p-*value**
Age, years, mean (SD)	56.5 (14.9)	53.6 (14.4)	60.2 (14.9)	0.004
Age≥ 60 years, *n* (%)	78 (45.1)	35 (36.1)	43 (56.6)	0.011
Male sex, *n* (%)	118 (68.2)	63 (65.0)	55 (72.4)	0.381
Diabetes, *n* (%)	42 (24.3)	15 (15.5)	27 (35.5)	0.004
Hypertension, *n* (%)	85 (49.1)	45 (46.4)	40 (52.6)	0.508
Hyperlipidemia, *n* (%)	108 (62.4)	62 (63.9)	46 (60.5)	0.765
Congestive heart failure, *n* (%)	5 (2.9)	4 (4.1)	1 (1.3)	0.386
Valvular heart disease, *n* (%)	2 (1.2)	1 (1.0)	1 (1.3)	1.000
Deep vein thrombosis, *n* (%)	2 (1.2)	0 (0.0)	2 (2.6)	0.192
**Previous vascular event**				0.191
None, *n* (%)	136 (78.6)	80 (82.5)	56 (73.7)	
TIA, *n* (%)	10 (5.8)	3 (3.1)	7 (9.2)	
Stroke, *n* (%)	23 (13.3)	13 (13.4)	10 (13.2)	
Coronary artery disease, *n* (%)	4 (2.3)	1 (1.0)	3 (4.0)	
Current smoker, *n* (%)	46 (26.6)	22 (22.7)	24 (31.6)	0.254
Follow-up time, years, mean (SD)	2.5 (1.7)	2.9 (1.7)	2.1 (1.5)	0.001
Index event	0.844
Acute ischemic stroke, *n* (%)	159 (91.9)	90 (92.8)	69 (90.8)	
TIA, *n* (%)	14 (8.1)	7 (7.2)	7 (9.2)	
**PFO features**				< 0.001
Small shunt, *n* (%)	73 (42.2)	19 (19.6)	54 (71.1)	
Moderate/Large shunt, *n* (%)	100 (57.8)	78 (80.4)	22 (29.0)	
Atrial septal aneurysm, *n* (%)	2 (1.2)	1 (1.0)	1 (1.3)	1.000
**Time metrics of PFO**				
Days from index event to microbubble test, median (IQR)	9 (7.3–21.9)	9 (7.3–14.6)	9 (7.3–21.9)	0.677
Days from microbubble test to PFO closure, median (IQR)		4 (3.7–21.9)		na
Days from index event to PFO closure, median (IQR)		13 (10.5–35.5)		
RoPE score, mean (SD)	5.5 (1.9)	5.9 (1.8)	5.0 (2.0)	0.001
RoPE score ≥ 6, *n* (%)	84 (48.6)	54 (55.7)	30 (39.5)	0.050
NIHSS at admission, median (IQR)	2 (1–6)	2 (0–5)	2 (1–7)	0.690
mRS at admission, median (IQR)	2 (1–3)	2 (1–3)	2 (1–4)	0.230
**Antithrombotic at long-term**				0.204
No antithrombotic, *n* (%)	1 (0.6)	1 (1.0)	0 (0.0)	
SAPT, *n* (%)	144 (83.2)	84 (86.6)	60 (79.0)	
DAPT, *n* (%)	20 (11.6)	9 (9.3)	11 (14.5)	
Warfarin, *n* (%)	3 (1.7)	0 (0.0)	3 (4.0)	
DOAC, *n* (%)	5 (2.9)	3 (3.1)	2 (2.6)	

PFOC, patent foramen ovale closure; SD, standard deviation; TIA, transient ischemic attack; IQR, interquartile range; RoPE score, Risk of Paradoxical Embolism Score; NIHSS, NIH Stroke Scale; mRS, modified Rankin Scale; SAPT, single-antiplatelet therapy; DAPT, dual antiplatelet therapy; DOAC, direct oral anticoagulant; and na, not applicable.

### 3.2. Baseline characteristics between the non-elderly and elderly groups

Demographic characteristics, comorbidities, and stroke severity were comparable between the PFOC and non-PFOC subgroups in both age groups (Table 2). However, similar to the characteristics of the total cohort, the PFOC subgroups in both age groups had more patients with moderate or large shunt size (PFOC vs. non-PFOC group, non-elderly group, 80.7% vs. 18.2%, *p* < 0.001; elderly group, 80.0% vs. 37.2%, *p* < 0.001), but a similar proportion of ASA compared to the non-PFOC subgroup. The RoPE score and the proportion of RoPE score ≥6 were similar between the subgroups of PFOC and non-PFOC in both age groups.

**Table 2:** Baseline characterization of patients with PFO closure and non-PFO closure in the non-elderly and elderly groups.

	**Non-elderly group (*****n*** = **95)**			**Elderly group (*****n*** = **78)**		
**Characteristics**	**PFOC (*****n*** = **62)**	**Non-PFOC (*****n*** = **33)**	***p*** **-value**	**PFOC (*****n*** = **35)**	**Non-PFOC (*****n*** = **43)**	***p*** **-value**
Age, mean ± SD, y	45.0 ± 9.4	46.1 ± 8.3	0.639	68.8 ± 7.4	71.0 ± 8.0	0.203
Male sex, *n* (%)	40 (64.5)	23 (69.7)	0.779	23 (65.7)	32 (74.4)	0.556
Diabetes mellitus, *n* (%)	9 (14.5)	10 (30.3)	0.118	6 (17.1)	17 (39.5)	0.056
Hypertension, *n* (%)	25 (40.3)	14 (42.4)	1.000	20 (57.1)	26 (60.5)	0.948
Hyperlipidemia, *n* (%)	40 (65.5)	21 (63.6)	1.000	22 (62.9)	25 (58.1)	0.849
Congestive heart failure, *n* (%)	4 (6.5)	1 (3.0)	0.655	0 (0.0)	0 (0.0)	na
Valvular heart disease, *n* (%)	0 (0.0)	0 (0.0)	na	1 (2.9)	1 (2.3)	1.000
Deep vein thrombosis, *n* (%)	0 (0.0)	2 (6.1)	0.118	0 (0.0)	0 (0.0)	na
Previous vascular event			0.261			0.222
None, *n* (%)	54 (87.1)	27 (81.8)		26 (74.3)	29 (67.4)	
TIA, *n* (%)	3 (4.8)	2 (6.1)		0 (0.0)	5 (11.6)	
Stroke, *n* (%)	5 (8.1)	2 (6.1)		8 (22.9)	8 (18.6)	
Coronary artery disease, *n* (%)	0 (0.0)	2 (6.1)		1 (2.9)	1 (2.3)	
Current smoker, *n* (%)	18 (29.0)	12 (36.4)	0.617	4 (11.4)	12 (27.9)	0.131
Index event			0.715			1.000
Acute ischemic stroke, *n* (%)	57 (91.9)	29 (87.9)		33 (94.3)	40 (93.0)	
TIA, *n* (%)	5 (8.1)	4 (12.1)		2 (5.7)	3 (7.0)	
PFO features			< 0.001			
Small shunt, *n* (%)	12 (19.4)	27 (81.8)		7 (20.0)	27 (62.8)	< 0.001
Moderate/Large shunt, *n* (%)	50 (80.7)	6 (18.2)		28 (80.0)	16 (37.2)	
Atrial septal aneurysm, *n* (%)	1 (1.6)	0 (0.0)	1.000	0 (0.0)	1 (2.3)	1.000
Time metrics of PFO						
Days from index event to microbubble test, median (IQR)	9 (8–18)	8 (8–25)	0.987	8 (8–18)	9 (8–19)	0.528
Days from microbubble test to PFO closure, median (IQR)	4 (4–18)		na	4 (4–19)		na
RoPE score, mean ± SD	6.8 ± 1.4	6.5 ± 1.7	0.389	4.3 ± 1.1	3.8 ± 1.2	0.071
RoPE score ≥6, n (%)	52 (83.9)	27 (81.8)	1.000	2 (5.7)	3 (7.0)	1.000
NIHSS at admission, median (IQR)	2 (0–5)	2 (0–7)	0.702	3 (1–5)	2 (1–7)	0.700
mRS at admission, median (IQR)	1 (1–3)	2 (1–4)	0.401	3 (1–4)	2 (1–4)	0.898

PFOC, patent foramen ovale closure; SD, standard deviation; TIA, transient ischemic attack; IQR, interquartile range; RoPE score, Risk of Paradoxical Embolism Score; NIHSS, NIH Stroke Scale; mRS, modified Rankin Scale; and na, not applicable.

### 3.3. Outcomes and subgroup analyses

The primary outcome occurred in 6 of 97 patients (6.2%) in the PFOC group and 13 of 76 patients (17.1%) in the non-PFOC group during a mean follow-up of 2.5 years (HR 0.35, 95% CI, 0.13–0.97, *p* = 0.043) (Table 3).

**Table 3:** Outcomes of recurrent cerebral ischemic events and all-cause mortality during the follow-up period.

**Recurrent cerebral ischemic event and all-cause mortality^a^**	**PFOC**	**Non-PFOC**	***p-*value**	**HR (95%CI)**	***p-*value for HR**
Total cohort, *n*/*N* (%)	6/97 (6.2)	13/76 (17.1)	0.042	0.35 (0.13–0.97)	0.043
Age < 60 years, *n*/*N* (%)	3/62 (4.8)	3/33 (9.1)	0.417	0.61 (0.11–3.37)	0.574
Age ≥60 years, *n*/*N* (%)	3/35 (8.6)	10/43 (23.3)	0.083	0.26 (0.07–1.01)	0.051

PFOC, patent foramen ovale closure.

a Recurrent cerebral ischemic event and mortality analysis by adjusted Cox regression test and adjusted for the RoPE score and NIHSS at admission.

**Figure 2:**
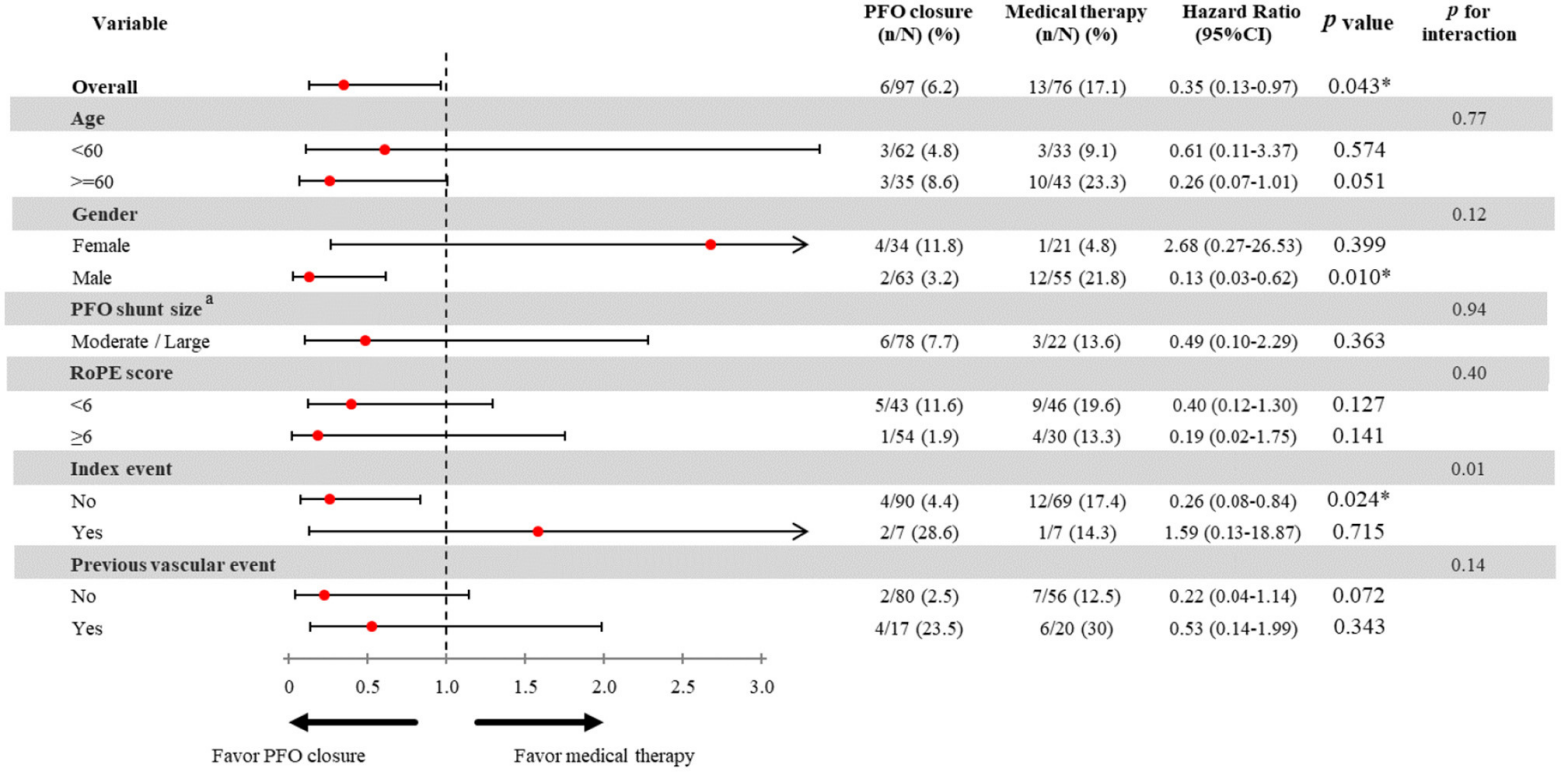
Effect of PFO closure on the risk of recurrent ischemic stroke. PFO, patent foramen ovale; RoPE, risk of paradoxical embolism score; TIA, transient ischemic stroke. ^a^The hazard ratio PFO closure on small PFO shunt size was not analyzed due to limited sample size. The incidence of the primary outcome in patients with small shunt size and PFOC was 0% in our study, and not available to perform analysis stratified by shunt size.

**Figure 3:**
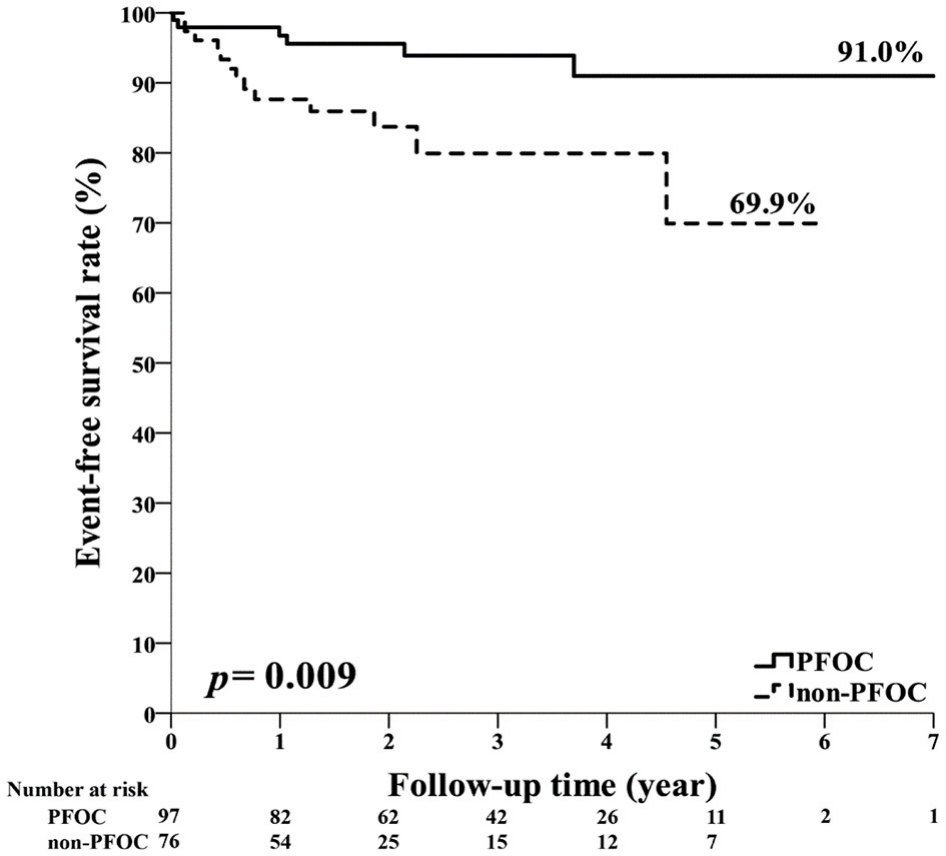
The rate of recurrent ischemic stroke between PFOC and non-PFOC.

In the subgroup analyses, male patients had a lower risk of the primary outcome (HR 0.13, 95% CI 0.03–0.62, *p* = 0.001), but the interaction *p*-value between the sex subgroups was not significant. Patients with an entry event of ischemic stroke had a significantly reduced risk of the primary outcome (HR 0.26, 95% CI 0.08–0.84, *p* = 0.024, *p*-interaction = 0.01). Compared with the non-PFOC group, the PFOC group had a numerically lower risk of recurrent ischemic stroke and all-cause mortality in both the elderly (HR 0.26, 95% CI 0.07–1.01, *p* = 0.051) and the non-elderly (HR 0.61, 95% CI 0.11–3.27, *p* = 0.574) groups, and the interaction *p*-value between the age subgroups was not significant (Figure 2). The Kaplan–Meier survival curve with the log-rank test of the total cohort showed that the PFOC group had a better event-free survival rate than the non-PFOC group (91.0% vs. 69.9%, *p* = 0.009) (Figure 3).

Compared to the non-PFOC group, more patients in the PFOC group had a favorable outcome at 180 days (89.7% vs. 77.6%, *p* = 0.05) (Table 4). By logistic regression, patients with PFO closure in the total cohort were more likely to have a favorable outcome at 180 days (OR 2.51, 95% CI 1.07–5.85, *p* = 0.034). Furthermore, patients with PFO closure had a numerically higher probability of a favorable outcome at 180 days in both the elderly (OR 2.09, 95% CI 0.76–6.25, *p* = 0.185) and the non-elderly (OR 2.00, 95% CI 0.47–8.58, *p* = 0.351) groups although it was not statistically significant. The median mRS at 180 days was not statistically different between the PFOC and non-PFOC groups in the total cohort and the non-elderly group. However, among the elderly group, the PFOC group had a lower median mRS at 180 days than the non-PFOC group (IQR 0–2 vs. 0–3, *p* = 0.002).

**Table 4:** Favorable functional outcomes and MRS at 180 days.

	**PFOC (*n* = 97)**	**Non-PFOC (*n* = 76)**	***p-*value**	**OR (95%CI)**	***p-*value for OR**
mRS at 180 days^a^					
Total cohort, median (IQR)	0 (0–1)	1 (0–2)	0.074		
Age < 60 years, median (IQR)	0 (0–1)	1 (0–1.5)	0.18		
Age ≥60 years, median (IQR)	1 (0–2)	1 (0–3)	0.002		
Favorable outcome at 180 days^b^					
Total cohort, *n* (%)	87 (89.7)	59 (77.6)	0.05	2.51 (1.07–5.85)	0.034
Age < 60 years, *n* (%)	58 (93.5)	29 (88.9)	0.443	2.00 (0.47–8.58)	0.351
Age ≥60 years, *n* (%)	29 (82.9)	30 (69.8)	0.180	2.09 (0.70–6.25)	0.185

mRS, modified Rankin Scale; IQR, interquartile range; PFOC, patent foramen ovale closure.

a mRS at 180 days analysis by the Mann-Whitney U-test.

b Favorable outcome at 180 days analysis by simple model logistic regression.

### 3.4. Safety outcomes

In 97 patients with PFO closure, five procedure-related adverse events (5.2%) and five new-onset periprocedural AF (5.2%) occurred, respectively (Table 5). Procedure-related adverse events comprised four with femoral hematoma and one with minimal pericardial effusion. Furthermore, the rate of procedure-related adverse events (non-elderly vs. elderly group, 3.2% vs. 8.6%, *p* = 0.348) and new-onset AF (non-elderly vs. elderly group, 4.8% vs. 5.7%, *p* = 1.000) did not differ significantly between two age groups.

**Table 5:** Safety outcomes from PFO closure.

	**PFOC (*n* = 97)**	**Non-elderly group (*n* = 62)**	**Elderly group (*n* = 35)**	***p-*value**
Device-related adverse event, *n* (%)	5 (5.2)	2 (3.2)	3 (8.6)	0.348
Periprocedural AF, *n* (%)	5 (5.2)	3 (4.8)	2 (5.7)	1.000

PFOC, patent foramen ovale closure; AF, atrial fibrillation.

## 4. Discussion

Our study, which included patients with PFO and CS or cryptogenic TIA, comprised 45.1% of patients who are 60 years or older. The main findings of this study were the following: (1) compared with the non-PFOC group, the risk of recurrent ischemic stroke and all-cause mortality in the PFOC group was significantly lower in the total cohort and numerically lower in both the non-elderly and elderly groups, (2) the safety outcomes of PFO closures were not different between the non-elderly and elderly groups, (3) more patients who received PFO closure had a favorable outcome at 180 days, and (4) patients with PFO closure in the elderly group had lower mRS at 180 days compared to those without PFO closure.

Previous randomized control trials have shown the efficacy of PFO closure in preventing future strokes (3, 5, 17–19) and all-cause mortality (18, 19). Based on the inclusion and exclusion criteria of these trials, it is generally suggested that PFO closure is reasonable in strictly selected patients who meet the following criteria: (1) CS, (2) large PFO shunt, (3) high RoPE score, and (4) age <60 years (4, 19).

Most studies did not include patients older than 60 years due to multiple potentially competing causes of stroke in the elderly population (3, 5, 17, 20, 21). However, in clinical practice, more than half of patients with AIS are older than 60 years. There is a high prevalence of PFO in elderly patients with CS (1). Increasing age is associated with a higher risk of recurrent ischemic stroke in patients with CS or cryptogenic TIA and PFO (7, 8). One reason is that the diameter of PFO and the prevalence of venous thrombosis increase with age, (22) and the risk of paradoxical emboli may increase accordingly (23). Additionally, elderly patients with PFO are more prone to right-to-left shunting due to a higher prevalence of sleep apnea (24). In our study, PFO closure numerically reduces the occurrence of recurrent stroke and all-cause mortality in patients above 60 years. Similarly, the DEFENSE-PFO trial enrolled patients up to 80 years of age and showed the efficacy of PFO closure in preventing recurrent stroke (18). A substudy of the DEFENSE-PFO trial indicates that the benefit of PFO closure is greater in elderly patients, even 70 years or older, than in younger patients (25). Our study is consistent with the DEFENSE-PFO trial and cohort studies (10, 25).

Although the primary outcome did not reach a statistical difference between the PFOC and non-PFOC groups in either the non-elderly or elderly group, patients with PFO closure in the elderly group had a lower mRS at 180 days than those without PFO closure. Because the follow-up after discharge was based on the telephone questionnaire but not the follow-up neuroimaging, we could not rule out the possibility that the family or the patient might not record mild ischemic events detrimental to the functional outcome. In addition, we could not rule out that the favorable functional outcome at 180 days might be caused by recurrent ischemic stroke and, more importantly, the baseline NIHSS score at admission. Because only 27 patients encountered an unfavorable functional outcome in our study, we cannot do further statistical analysis to provide robust evidence. However, considering the efficacy and safety profiles of PFO closure in our study, our results suggest that elderly patients with CS and PFO might also benefit from PFO closure. Given that the rate of recurrent strokes was low in both PFOC and non-PFOC patients, we could not rule out that mRS at 180 days was more likely driven by the severity of the index stroke instead of the potential benefit of PFO closure.

The rates of procedure-related adverse events and new-onset periprocedural AF were low, and there was no significant difference between the non-elderly and elderly groups. The rate of new-onset AF in the elderly group was 5.7% compared with previous studies in the population aged <60 years (3, 5, 17–19). Consistent with previous studies in the elderly population (10, 11), PFO closure was a safe procedure in the elderly group, with a low rate of device-related adverse events and new-onset AF. Advanced age may not be considered a limitation to PFO closure in elderly patients with CS and PFO.

Ischemic stroke secondary to PFO can occur due to thrombus formation around the PFO or paradoxical embolism from the venous system through the PFO due to the right-left shunt (26). As a result, a large shunt or the presence of ASA are generally considered high-risk features of PFO. However, in clinical practice, the results of the etiological survey of stroke are usually obscure. For example, a cortical embolic stroke with a small PFO shunt that lacks high-risk anatomical features or has a low RoPE score can raise the difficulty of determining the causal relationship between PFO and the index stroke. Four large trials recruited PFO patients with CS without requiring high-risk anatomical characteristics (3, 17, 20, 21). Two of them still showed a significant reduction in recurrent stroke possibly due to more strict criteria of CS (3, 17). Our study also included patients with non-strictly selected PFO which comprised a small shunt of 42.2% and ASA of only 1.2%. The closure of the PFO still significantly reduced the risk of the composite of recurrent ischemic stroke and all-cause mortality. However, because the incidence of the primary outcome in patients with small shunt size and PFOC was 0%, we were unable to perform an analysis stratified by shunt size.

Because the RoPE score is a composite index estimated by six clinical characteristics, including age, smoking, cortical infarct on imaging, hypertension, diabetes, and history of stroke or TIA, (14) there were hyperbolic correlations between the RoPE score and the six characteristics. Thus, we included the RoPE score instead of individual characteristics as an adjusted variable in the model. Moreover, previous studies showed that baseline NIHSS is associated with the recurrence of ischemic stroke and mortality (27, 28). Finally, the RoPE score and baseline NIHSS at admission were selected as adjusted variables in the multivariable regression model. We also retrospectively applied the PASCAL classification system in the study cohort according to the RoPE score and the high-risk characteristics of PFO (29). The results showed that 41.6%, 48.0%, and 10.4% were classified as unlikely, possible, and probable groups, respectively. PFO closure was performed in 33.3%, 66.3%, and 100.0% of the unlikely, possible, and probable groups, respectively. Although the PFOC group had a higher frequency of moderate-large shunt and higher RoPE score, the use of PFO closure in our study was not entirely in accordance with the PASCAL classification system. For example, in elderly patients with PFO closure, 20% had a small PFO shunt, and the mean RoPE score (SD) was 4.3 (1.1). However, according to the statements in the European position document, no single clinical, anatomical, or imaging characteristics are sufficient to make a quantitative estimate of the probability of a causal role (2). The indication of PFO closure should be individualized based on considering the potential causative role of PFO in the index stroke instead of the exclusive age.

Regarding the timing to perform the PFO closure, previous large-scale studies tend to postpone the procedure to 80–100 days after the onset of acute stroke (3, 5). However, in real-world practice, patients might lose follow-up in the 3–4 months follow-up period. Furthermore, the postponement of the procedure may carry a risk of stroke recurrence in the follow-up period. Patients in our study underwent PFO closure much earlier than in other studies after the index event. We performed a microbubble test and PFO closure with a median of 9 and 13 days after the index stroke, respectively. The favorable profile of efficacy and safety in our study indicates that PFO closure in the acute stage of stroke is reasonable management and might help reduce recurrent ischemic events in the long term.

Our study had several limitations. First, this is not a randomized controlled study, so we could not avoid the selection bias and the imbalance between treatment groups in real-world practice. Second, the follow-up periods varied among patients, so there could be bias in estimating recurrent ischemic events. Third, the number of patients was insufficient for powered subgroup analyses. Fourth, for those patients in the non-PFOC group, diagnostic cardiac catheterizations were not performed, so a rare chance of diagnosis other than PFO could not be ruled out.

## 5. Conclusion

PFO closure significantly reduced the risk of a composite of recurrent ischemic stroke and all-cause mortality compared to medical therapy alone in our total cohort. Furthermore, elderly patients with PFO closure had a more favorable functional outcome at 180 days than those without PFO closure. In addition, our study also showed that PFO closure might be safe and appropriate for patients with CS and PFO in the acute stage of stroke. Further randomized trials or large-scale registries of elderly patients with CS and PFO are needed to clarify the efficacy and safety of PFO closure in elderly populations.

## Data availability statement

The raw data supporting the conclusions of this article will be made available by the authors, without undue reservation.

## Ethics statement

The studies involving human participants were reviewed and approved by the Institutional Review Board (IRB) of Taichung Veterans General Hospital (CG19339B). Written informed consent from the patients/participants or patients/participants' legal guardian/next of kin was not required to participate in this study in accordance with the national legislation and the institutional requirements.

## Author contributions

P-LC, C-SW, and Y-CF: study design, advisor on writing, manuscript editing, and interpretation of results. J-AH, Y-HW, and N-CL: data collection and interpretation of results. C-YH: data analysis and statistical computation. All authors reviewed the results and approved the final version of the manuscript.
